# Experimental Investigation of Wind Pressure Characteristics for Cladding of Dome Roofs

**DOI:** 10.3390/ma14185266

**Published:** 2021-09-13

**Authors:** Dong-Jin Cheon, Yong-Chul Kim, Jong-Ho Lee, Sung-Won Yoon

**Affiliations:** 1 Department of Architecture, Seoul National University of Science & Technology, 232, Gongneung-ro, Nowon-gu, Seoul 01811, Korea; djs0716@seoultech.ac.kr (D.-J.C.); structure@seoultech.ac.kr (J.-H.L.); 2 Department of Architecture, Tokyo Polytechnic University, Atsugi 243-0297, Japan; kimyc@arch.t-kougei.ac.jp

**Keywords:** dome roof cladding, wind tunnel test, pressure distribution

## Abstract

Cladding for dome roofs is often made of membrane materials that are light and easy to install. Due to these characteristics, wind damage to dome roof cladding is very common. In particular, open or retractable dome roofs are prone to wind damage because of inadequacies in wind load calculations. In this study, the wind pressure characteristics of a dome with a central opening were investigated. Wind tunnel tests were performed, and the pressure distribution was investigated by analyzing external and internal pressure coefficients. Based on the experimental results, the peak net pressure coefficients for the cladding design of a dome roof with a central opening were proposed. For the external peak pressure coefficients, the values of leeward regions were similar despite height–span ratios and turbulence intensity values. For the internal peak pressure coefficients, negative pressure was dominant, and the coefficients were not significantly affected by changes in height–span ratio. This tendency locally increased the negative peak net pressure, in which the load acts in the upward direction, and relatively significantly increased the positive peak net pressure, in which the load acts in the downward direction.

## 1. Introduction

Dome roofs are widely used in large-space structures for their structural efficiency and economic advantages. In particular, membranes, which are easy to handle because of their low weight and convenient construction, are widely used as cladding materials for roofs over large structures. However, membrane dome roofs are sensitive to wind loads because of their long span structure and lightweight members. Consequently, numerous studies have evaluated the pressure distribution of dome roofs. For example, Uematsu et al. [1] investigated the pressure distribution in domes with various rise-span ratios (hereinafter referred to as *f*/*D*) and wall height–span ratios (hereinafter referred to as *H*/*D*). Their research confirmed that changes in *f*/*D* have a greater impact on changes in the pressure distribution than those in *H*/*D*. Noguchi and Uematsu [2] also examined the wind pressure characteristics through wind tunnel tests for domes with varying *f*/*D* and *H*/*D* values; based on their results, they proposed pressure coefficients for the cladding and frame design by the zone of the dome roof. The current Japanese wind load code (AIJ-RLB (2015)) uses the values reported in their research. Letchford and Sakar [3] investigated the mean and fluctuating pressure distribution of a dome roof according to the surface roughness. Their analysis showed that a rough surface reduced suctions over the apex of the dome and increased suctions in the wake region. Cheng and Fu [4] conducted wind tunnel tests for various Reynolds numbers in smooth flow and turbulent boundary layer flow, and they found that the pressure distribution was stable when the Reynolds number was between 1.0 × 10^5^ and 2.0 × 10^5^ for the turbulent boundary layer flow. Kharoua and Khezzar [5] investigated the turbulent flow around a dome through a computational fluid dynamics (CFD) simulation in smooth flow and turbulent flow. Their analysis confirmed that the turbulent effect in the wake region was greater in turbulent flow than in smooth flow. Sun and Qiu [6] investigated the characteristics of the wind pressure spectrum according to various *f*/*D* and *H*/*D* values for a dome roof, and they subsequently proposed a wind pressure spectrum model for each region. Recently, the number of retractable dome roof structures whose operation is unaffected by weather has been increasing worldwide. Because these structures can adjust to open, semi-open, and enclosed roofs, wind loads for various roof types should be considered. However, limited research has been conducted on retractable dome roof structures. Liu et al. [7] analyzed the net pressure coefficient of a retractable dome roof structure via wind tunnel experiments and large eddy simulations (LES), and they investigated the effect of wind based on the conditions of the roof. The results of their study demonstrated that the LES is effective for estimating wind loads in complex turbulent flows according to the Reynolds number. Kim et al. [8] conducted a study to develop a component that automatically estimates the wind load and conducts a structural analysis of retractable large space structures. A method to automatically derive structural analysis results was developed by linking the conversion of a structural analysis model through the structural analysis automation step in real time with the allocation of the wind load to the structure according to its shape. The two studies mentioned above employ structures with a unique shape, and thus, the distribution characteristics of the wind pressure according to the roof shape cannot be easily obtained, rendering the studies insufficient for general open or retractable dome roof shapes.

Extensive damage can be caused to a dome roof due to the wind in the actual open or retractable dome roofs. Cheon et al. [9] investigated dome roof damages worldwide. Table 1 summarizes some notable examples of wind-damaged roof membrane cladding. Six out of the eight cases involved open or retractable roofs. Most damages were caused by unexpected strong winds and inappropriate wind load calculations during the design process. In the case of closed dome roofs, many studies have been conducted and a wind load code has been established. In contrast, there is no code for open dome roofs and it is difficult to find any studies that can be referenced in the design process [9,10,11].

In general, structural types or members are often determined by referring to codes in the basic design stage [9,12]. Therefore, in this study, wind tunnel tests were performed on a dome with an opening in the center, and the pressure distribution was investigated through analyses of the external and internal pressure coefficients. In addition, the applicability of the current wind load code was examined through a comparison with the Japanese wind load code (AIJ-RLB (2015)). Based on this comparison and the characteristics of the pressure distribution, peak pressure coefficients were proposed for the cladding design applicable to central open dome roofs.

## 2. Wind Tunnel Tests

### 2.1. Model Details

**Figure 1:**
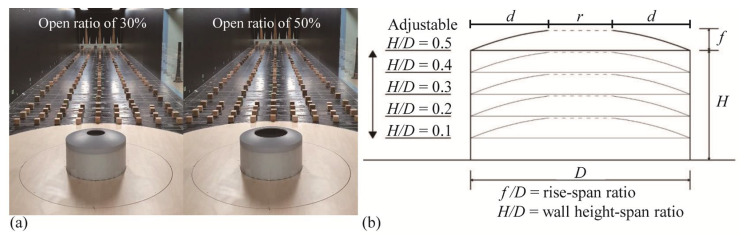
Photo of test model in wind tunnel and section of model: (**a**) models and (**b**) section of model.

The model used in the experiment (Figure 1a) was comprised of acrylic, and the roof of the central open dome was simulated. Figure 1b shows a section of the model, where *f*, *H*, and *D* denote the rise of the dome roof, wall height, and span length, respectively. In this study, a length scale of 1/150 was adopted, and the blockage rate was a maximum of 2.0%. Thus, data correction was not required. In terms of specific values, *f*, *H*, and *D* are 0.04 m, 0.04–0.2 m, and 0.4 m, respectively—in full scale, these values are 6 m, 6–30 m, and 60 m, respectively. The open ratio was defined based on the span length *D* of the model. For a total span length of 0.4 m and an open ratio of 30%, the model was produced with 0.12 m of the center of the dome open; for an open ratio of 50%, 0.2 m was open. Here, *H* was adjusted by 0.04 m using a turntable with adjustable heights to conduct the test. The specifications of the model are summarized in Table 1.

**Figure 2:**
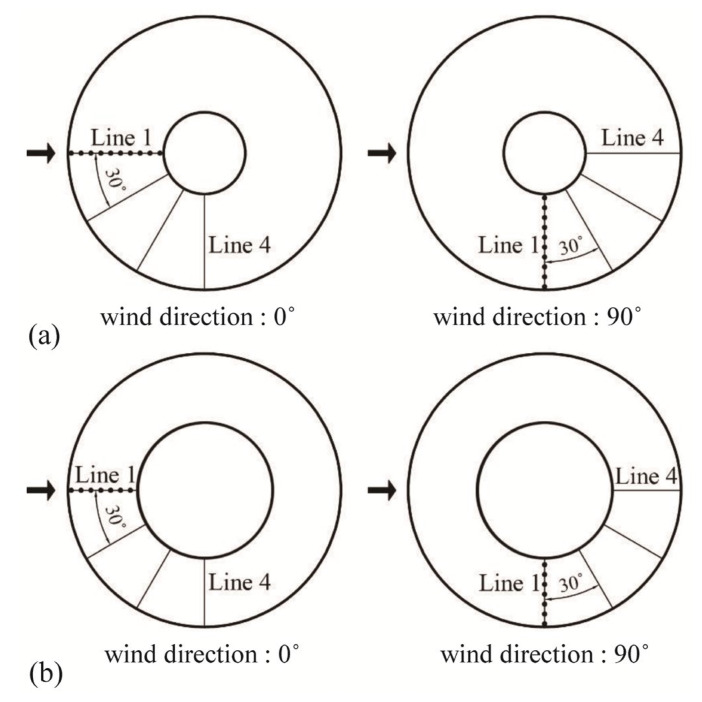
Pressure taps and wind direction: (**a**) open ratio = 30% and (**b**) open ratio = 50%.

Pressure taps were installed in four lines at 30° intervals on all external and internal sides of the roof surface. Ten pressure taps were installed per line for an open ratio of 30%, for a total of 80 taps. Seven pressure taps were installed per line with an open ratio of 50%, for a total of 56 taps. Because a spherical dome roof shows symmetrical values with respect to the centerline, the wind direction was adjusted to organize the data into a total of seven lines from windward line 1 to leeward line 7. As shown in Figure 2, when the wind direction is 90°, line 4 becomes line 7; further, line 4 at wind direction of 0° and line 1 at wind direction of 90° represent a line in the same position. The mean value of two data points was used. 

### 2.2. Approaching Flow Characteristics and Data Acquisition

**Figure 3:**
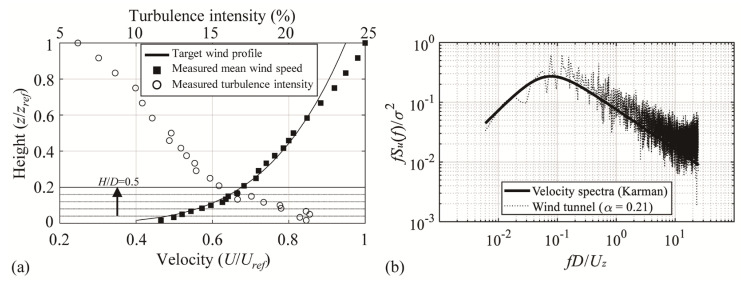
Characteristics of oncoming flow: (**a**) mean velocity and turbulence intensity profiles and (**b**) power spectra of velocity fluctuation at *z* = 0.24 m.

Wind tunnel tests were conducted in a large boundary layer wind tunnel with a width of 2.2 m and height of 1.8 m at Tokyo Polytechnic University, Japan. Figure 3a shows the vertical profiles of the mean wind velocity and turbulence intensity. An urban topography was assumed, and the target power-law exponent α of the average wind speed profile was set to 0.21. The turbulent boundary layers were reproduced using various spires and roughness blocks. The mean wind velocity and turbulence intensity varied depending on the wall height *H*, and the mean wind velocity at the maximum height *H* + *f* of the roof for each model was used to define the pressure coefficient. Assuming a wind speed scale at 1/3, for the model with *H*/*D* = 0.5, the mean wind velocity was 8.9 m/s and turbulence intensity was 15.4%. Figure 3b shows the power spectrum of the fluctuating wind speed at the roof height of the model *H*/*D* = 0.5 (*z* = 0.24 m), which is similar to the Karman spectrum. Considering the time scale obtained by the length scale and the wind speed scale, that is, (1/150)/(1/3) = 1/50, each pressure record was sampled for 12 s, which is equivalent to 10 min in full scale. The sampling frequency was 1000 Hz. In total, ten samples (12 × 10 = 120 s) were recorded in the pressure coefficient analysis for each *H*/*D*. All pressures were measured simultaneously using a multi-channel pressure system. The experimental conditions are summarized in Table 2.

**Figure 4:**
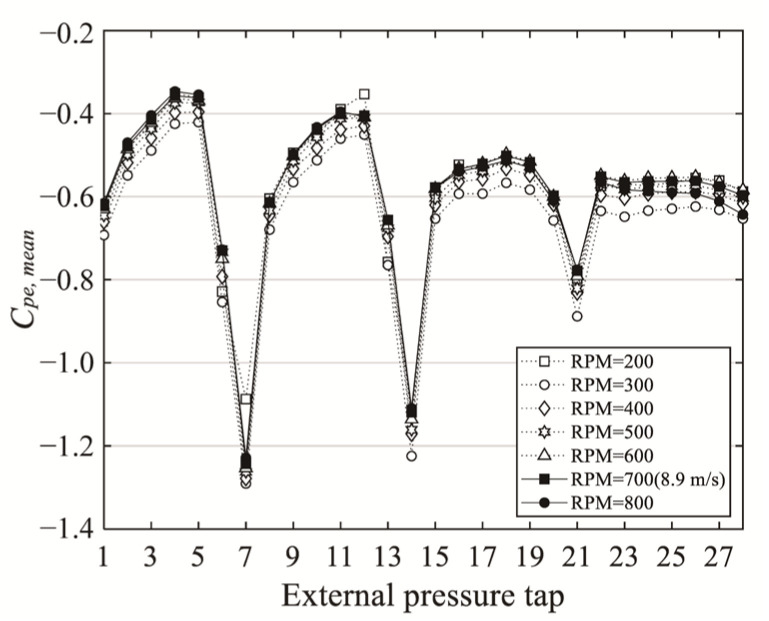
Distribution of the external mean pressure coefficients according to various wind velocities.

The Reynolds number was defined using the span length *D* and the mean wind velocity at the peak height of the roof, and the initial test was conducted to determine a constant Reynolds number with stable pressure coefficient values. Figure 4 shows the mean pressure coefficient of *H*/*D* = 0.5 measured for various wind velocities. When the wind velocity exceeded 8.9 m/s, the mean pressure coefficient could be confirmed to be constant. In this study, the Reynolds number varied from 1.8 × 10^5^ to 2.2 × 10^5^. According to previous studies of the dome roof, it was found that when the Reynolds number is more than 1.0 × 10^5^, the location of the separation does not change and the wind pressure is stable [4].

### 2.3. Pressure Coefficient Definitions

In this study, the pressure coefficient was calculated using the following Equations.

(1) $$C_{P,i}=\frac{P_{i}-P_{pitot}}{q_{H+f}}$$

(2) $$C_{P,mean}=\frac{1}{N}\overset{N}{\underset{i=1}{\sum }}C_{p,i}$$

(3) $$C_{P,rms}=\sqrt{\frac{1}{N}\sum_{i=1}^{N}{\left(C_{p,i}-C_{p,mean}\right)}^{2}}.$$

The external and internal pressure coefficients were calculated using Equation (1), where *P_i_* is the pressure at each pressure tap located on the model roof; *P_pitot_* is the pressure at the pitot tube installed 1.2 m above the wind tunnel floor; and *q_H+f_* is the velocity pressure at the peak height of the roof for each model. The mean and fluctuating pressure coefficients were calculated using Equations (2) and (3), respectively, and the peak pressure coefficients were defined as the minimum and maximum values for each *C_p,i_*. The pressure coefficient was calculated for each sample corresponding to an actual time of 10 min, and the mean value of a total of ten samples was used. 

To increase the reliability of the statistics, the Best Linear Unbiased Estimator (BLUE) method was used to estimate the peak pressure coefficient. The mean values of ten ensembles were compared with the extreme values obtained through the BLUE method; the extreme values were observed to be approximately 10% larger, but the fluctuation trends of the absolute values were similar. The wind load code (AIJ-RLB (2015)) used for the comparison was the average of ten ensembles. Therefore, to obtain an accurate comparison, ten ensemble average values were analyzed [13,14,15]. Further, the moving average time was 1 s, which was the same as that of the wind load code.

## 3. Results and Discussions

The analysis was conducted based on lines 1 and 7 of the centerline showing distinct changes. Line 1 was defined as windward, and line 7 was defined as leeward. The magnitude of the wind pressure coefficient was expressed in absolute values. Furthermore, because the open ratios of 30% and 50% were similar, the analysis was mainly conducted based on the latter value.

### 3.1. External and Internal Pressure Distribution on the Roof Surface

**Figure 5:**
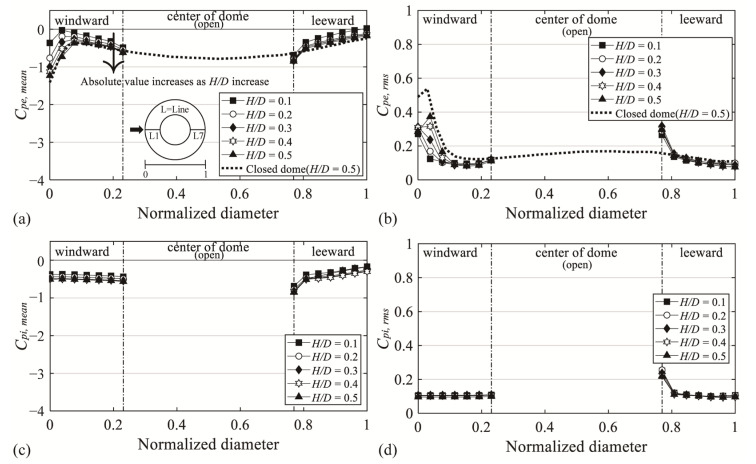
Variation of mean and fluctuating pressure coefficient depending on *H*/*D*: (**a**) *C_pe,mean_*, (**b**) *C_pe,rms_*, (**c**) *C_pi,mean_*, and (**d**) *C_pi,rms_.*

Figure 5a,b shows the mean and fluctuating pressure coefficients (*C_pe,mean_* and *C_pe,rms_*) for all *H*/*D* values with an open ratio of 50%. The x-axis represents the diameter normalized with the dome span length *D* and the pressure tap distance. Here, “0” indicates the edge of the windward roof, “1” denotes the edge of the leeward roof, and the y-axis represents the respective pressure coefficient. As the flow moved toward the leeward side, various changes occurred because of the separation, reattachment, and the boundary layer of the dome surface. As seen in Figure 5a, the absolute value of *C_pe,mean_* changes rapidly due to separation when the normalized diameter of the windward region is approximately 0–0.15. The decreasing absolute values started to increase again at a normalized diameter of 0.04 (Tap #2) for *H*/*D* = 0.1 and at 0.15 (Tap #5) for *H*/*D* = 0.5. This is because with the impact of the boundary layer formed on the dome surface, reattachment occurred again around the applicable positions. Compared with those of the taps at a normalized diameter of 0.23, the absolute values increased at a normalized diameter of 0.77, which corresponds to the roof edge of the open space, because the flow that deviated from the windward roof surface was separated at the corresponding location. After separation, the absolute value gradually decreased. The flow is assumed to not be along the dome roof surface, because reattachment does not occur, owing to the separation and shape of the roof. This phenomenon can also be observed in *C_pe,rms_*. Figure 5b shows *C_pe,rms_*; the absolute values are larger than those in the other regions at the normalized diameters of 0–0.15 for the windward side and 0.77 for the roof edge of the open space, owing to the effect of separation. For comparison, the dotted lines in Figure 5a,b represent *C_pe,mean_* and *C_pe,rms_* of *H*/*D* = 0.5 for a closed dome roof. The values and trends of the normalized diameters of 0–0.15 closely resemble those of the central open dome. However, in the region after reattachment, no notable change is observed compared with the central open dome. Figure 5c,d show the mean and fluctuating pressure coefficients (*C_pi,mean_* and *C_pi,rms_*) for the internal roof surface. The increase in the absolute values of *C_pi,mean_* and *C_pi,rms_* at the normalized diameter of 0.77 (i.e., roof edge of open space) is assumed to be caused by the separation of the same deviated flow as that in the external roof surface. The absolute values of all other regions were similar.

**Figure 6:**
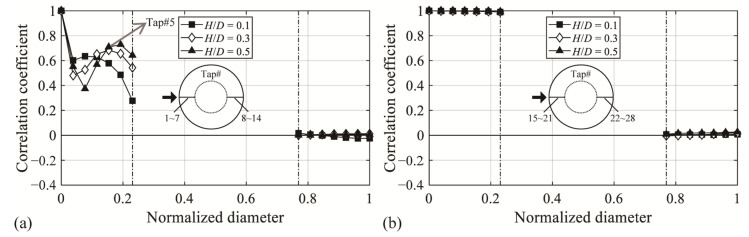
Cross-correlation coefficients of the pressure fluctuations: (**a**) external cross-correlation coefficient and (**b**) internal cross-correlation coefficient.

Figure 6a shows the cross-correlation coefficients for *H*/*D* = 0.1, 0.3, and 0.5, calculated based on Tap #1 located at the roof edge on the windward side with an open ratio of 50%. In the analysis, the reference pressure tap, Tap #1, was affected by the direct turbulence of the oncoming flows and separation. This pattern reflects the results of previous studies on closed domes with the same or similar *f*/*D* values [4,6,16]. Based on *H*/*D* = 0.5, the correlation coefficient rapidly decreased at Tap #2 and Tap #3. This was because as the flow moved leeward, the direct effect of the oncoming flow decreased, and the effect of the vortices owing to separation increased. The cross-correlation coefficient at Tap #4 and Tap #5 rapidly increased because with the movement in the flow, the space containing vortices gradually shrank owing to the roof shape and the effect of reattachment. The correlation coefficient of Tap #6 was similar to that of Tap #5 because this region was after the reattachment. The correlation coefficient decreased again because of the impact of boundary layer on the dome roof surface. From these results, with *H*/*D* = 0.5, reattachment occurs around the Tap #5 region, which is similar to the reattachment region defined in the mean and fluctuating pressure coefficients in Figure 5. In addition, the leeward correlation coefficient was independent of the windward correlation coefficient, although it was still affected by separation. This is believed to be because the leeward region was subjected to the effect of separation without the direct impact of the oncoming flows, compared with the case of windward flows. Figure 6b shows the cross-correlation coefficients of the interior roof surface, calculated based on Tap #15 located at the roof edge on the windward side. The windward region, being unaffected by the flow, has the same correlation coefficient at all areas, as shown by *C_pe,mean_*. By contrast, the leeward side has no relation with the windward side, similar to that in Figure 6a; this is because the internal roof is also affected by the separation of the deviated flow.

**Figure 7:**
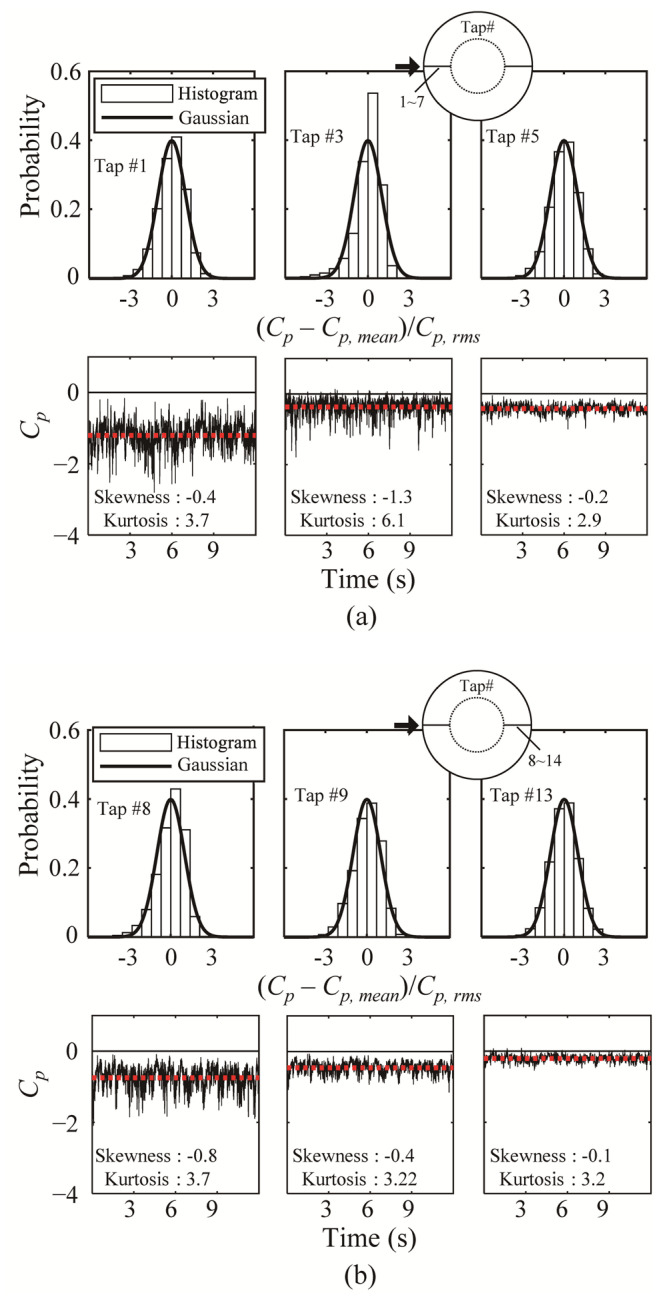
Histogram and time history of the pressure coefficient on external roof surface: (**a**) windward and (**b**) leeward regions.

**Figure 8:**
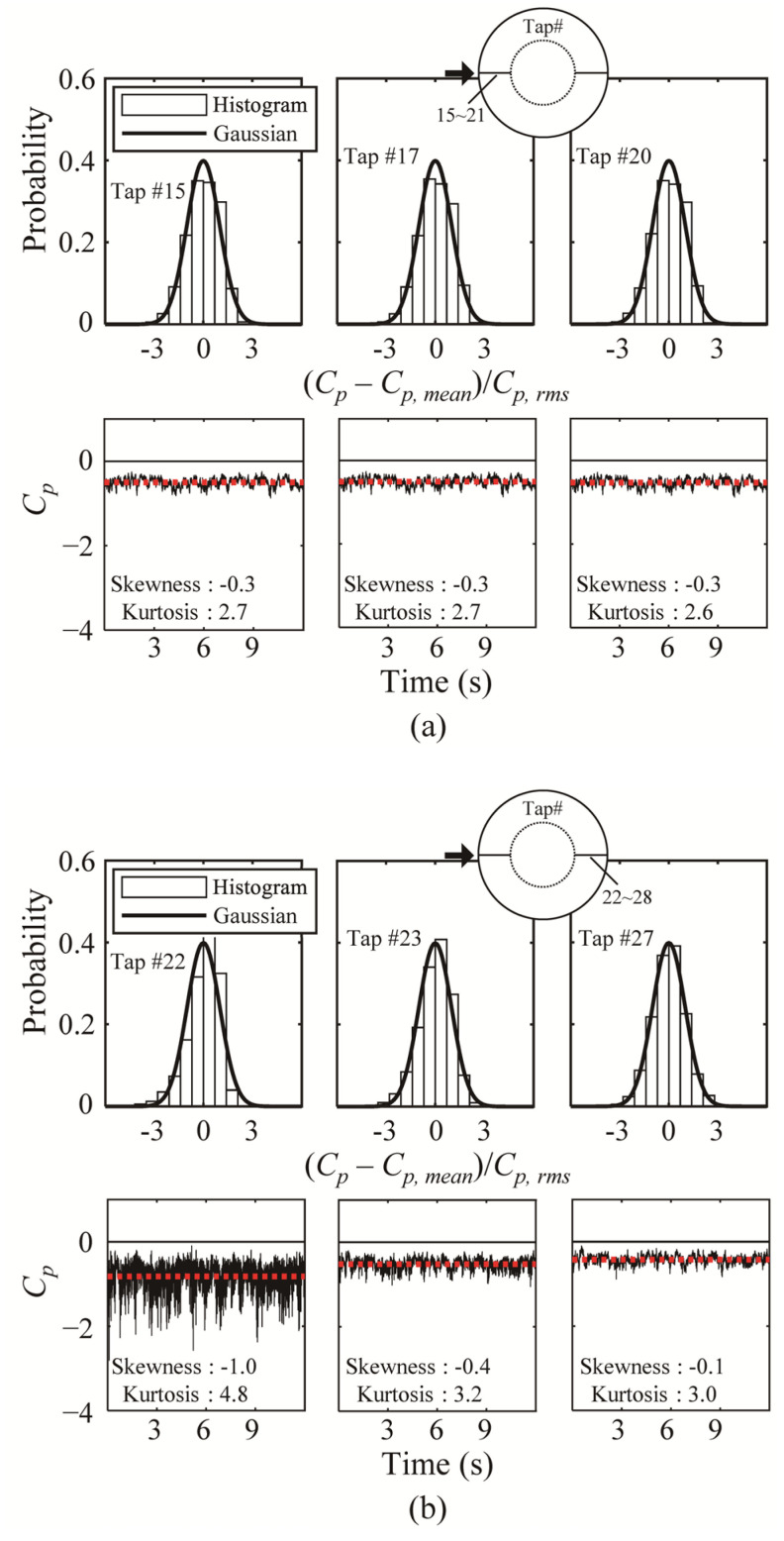
Histogram and time history of the pressure coefficient on internal roof surface: (**a**) windward and (**b**) leeward regions.

Figure 7 and Figure 8 show histograms of the probability distributions for some windward and leeward external and internal pressure taps, and the time series of the pressure coefficients. Here, *H*/*D* = 0.5, which has the largest absolute value, was used as a representative case. The time series of the pressure coefficients was obtained by selecting one random sample from among 10 samples. In the histogram, the x-axis represents the normalized pressure coefficient; the Gaussian distributions are shown to compare the characteristics of the probability distribution. In addition, the mean (dotted line), skewness and kurtosis values are expressed in the pressure coefficient time series. In general, in the area affected by separation, the roof pressure coefficients exhibit non-Gaussian characteristics with absolute values of the skewness and kurtosis of 0.5 and 3.5 or greater, respectively [17,18,19].

The histogram of Tap #1 in Figure 7 exhibits a shape similar to that of the Gaussian distribution; in addition, in the time histories, the mean value is larger than that of the other taps. However, the time history plot has a symmetrical shape around the mean value; this is because of the direct effect of the oncoming flows and the effect of separation, as described above. For Tap #3, the absolute values of skewness and kurtosis increase sharply compared with those of Tap #1. Accordingly, the histogram exhibits clear non-Gaussian characteristics, and the time series shows intermittent and distinct negative spikes. This phenomenon occurs because the direct impact of the oncoming flows decreases as the flow moves leeward, as described above. Tap #5 corresponds to the regions of reattachment; after reattachment, Gaussian characteristics are observed, with increasing absolute values of the pressure coefficients. Tap #8 exhibits clearer non-Gaussian characteristics than those of the tap affected by windward separation. Additionally, in the time series, the mean value of the pressure coefficients is slightly smaller than that of Tap #1; however, there is no effect of positive pressure, and the frequency and intensity of the negative spikes are larger than those of Tap #1. The absolute values of the pressure coefficients at the pressure taps after Tap #8 decrease gradually, exhibiting Gaussian characteristics. However, the absolute values are smaller than those of the tap in the windward reattachment region. This phenomenon also arises because reattachment does not occur, owing to the separation effect and shape of the roof, resulting in the absence of flow along the dome roof surface, as explained earlier.

Figure 8 shows the histogram and time series of the internal pressure taps. For the windward region, Gaussian characteristics are observed without any special features, and the histogram, mean, skewness and kurtosis are all similar. By contrast, Tap #22 exhibits non-Gaussian characteristics similar to Tap #8 in Figure 7; in other words, it is affected by the same separation as that in the external surface of the roof. Tap #23 and 27 exhibit Gaussian characteristics with a decreased pressure coefficient, similar to that in the case of external surface of roof. However, compared with the case of external surface, the absolute value of mean is slightly larger—this is because reattachment occurs due to the shape of the roof and is affected by the boundary layer on the dome surface.

### 3.2. Negative External Peak pressure Coefficients on the Roof Surface

**Figure 9:**
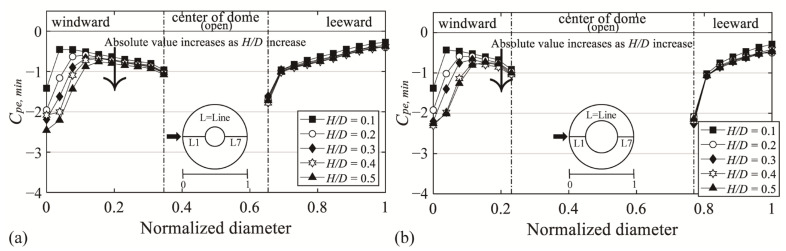
Variation of *C_pe,min_* depending on *H*/*D* of the central open dome: (**a**) open ratio = 30% and (**b**) open ratio = 50%.

Figure 9 shows the negative external peak pressure coefficient (*C_pe,min_*), with the x-axis denoting the normalized diameter and the y-axis denoting *C_pe,min_*. Notably, the absolute values for all *H*/*D* values are similar regardless of changes in *H*/*D* in the region where the separation of deviated flow occurs, unlike in the windward region where the absolute values increase with *H*/*D*. *C_pe,min_* ranges from −1.3 to −2.3 on the windward region; on the leeward region, it ranges from −1.6 to −1.8 and −2.1 to −2.3 at the open ratios of 30% and 50%, respectively. This occurs because the flow characteristics became similar, owing to the impact of the boundary layer formed on the dome roof surface after reattachment.

**Figure 10:**
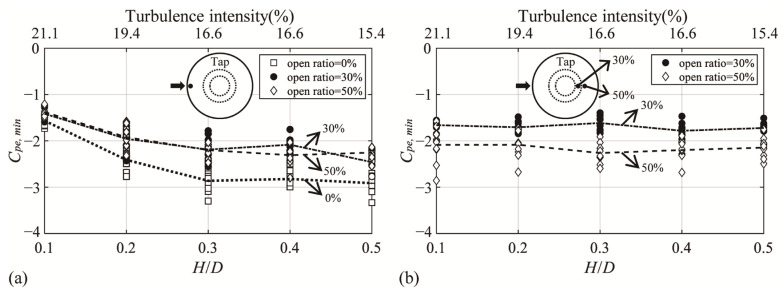
*C_pe,min_* according to the turbulence intensity (tap at the location affected by separation): (**a**) windward region and (**b**) edge of the roof on the center of the dome.

Figure 10 shows *C_pe,min_* according to changes in *H*/*D* and the turbulence intensity in two taps affected by separation. The x-axis expresses the *H*/*D* and turbulence intensity values for each *H*/*D*. In the figure, *C_pe,min_* is plotted for each of the 10 samples corresponding to the actual time of 10 min, and the dotted line represents the mean of 10 *C_pe,min_* values for each open ratio. For the windward tap, the data for the closed dome roof are also shown. As the turbulence intensity decreases, flow separation increases [3]. Thus, a decrease in turbulence intensity (increase in *H*/*D*) on the dome roof increases the reattachment distance. Further, as the size of the vortices generated by separation increases, the absolute values increase (Figure 10a). As *H*/*D* increases, the turbulence intensity decreases, and the absolute values increase accordingly. However, the *C_pe,min_* values at the roof edge tap in the open space are similar (Figure 10b). Therefore, the flow becomes constant, irrespective of changes in *H*/*D*. The absolute values of the open ratio of 50% are larger than those of 30%. This is presumably because the region affected by the boundary layer shrinks and the amount of incoming air increases as the open ratio increases.

**Figure 11:**
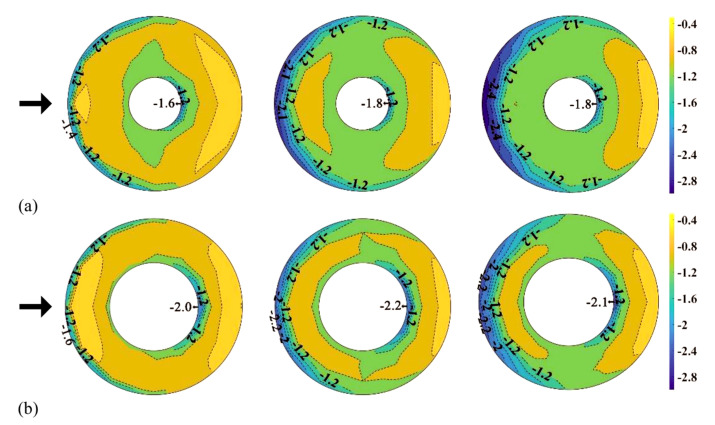
Distribution of *C_pe,min_*(*H*/*D* = 0.1, 0.3 and 0.5): (**a**) open ratio of 30% and (**b**) open ratio of 50%.

Figure 11 shows the distribution of *C_pe,min_* on the external surface of the central open-dome roof as a contour. In the roof with an open ratio of 50%, compared to the roof with an open ratio of 30%, the region affected by the separation of the deviated flow is more widely distributed. Thus, the absolute values are also larger. All other regions showed similar absolute values.

### 3.3. Positive External Peak Pressure Distribution on the Roof Surface

**Figure 12:**
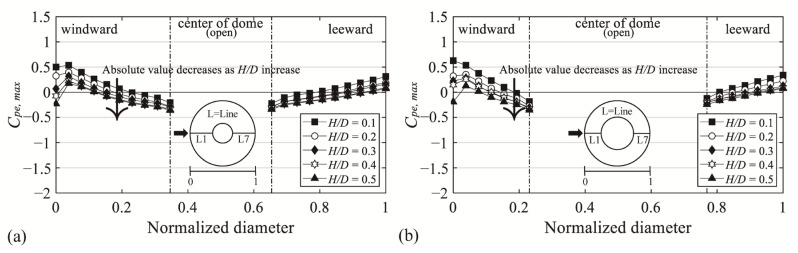
Variation of *C_pe,max_* depending on *H*/*D* of the central open dome: (**a**) open ratio = 30% and (**b**) open ratio = 50%.

Figure 12 shows the positive external peak pressure coefficient (*C_pe,max_*), with the x-axis as the normalized diameter and the y-axis as *C_pe,max_*. Compared with the values of a closed dome roof, the absolute values are similar in all regions without significant differences, unlike the case of *C_pe,min_*. Overall, as *H*/*D* increases, the absolute values decrease, and the region affected by the negative pressure broadens. This matches the results reported by Letchford and Sarkar [3] and Cheng and Fu [4]. The effect of surface roughness (increased turbulence intensity) moves the separation point thereby reducing the suction at the center of the dome roof surface. As in the results reported by Noguchi and Uematsu [2] and Kim et al. [20], the negative pressure dominates because of the low *f*/*D* value (*f*/*D* = 0.1); thus, the absolute values and changes of the coefficient were small compared to those of *C_pe,min_*. Because of the separation of the flow at the roof edge of the open space and the geometry of the roof, there was no effect of positive pressure.

**Figure 13:**
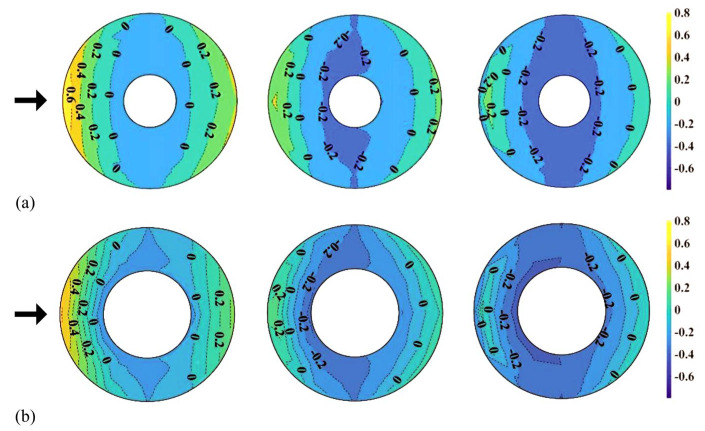
Distribution of *C_pe,max_*(*H*/*D* = 0.1, 0.3 and 0.5): (**a**) open ratio of 30% and (**b**) open ratio of 50%.

Figure 13 shows the distribution of *C_pe,max_* on the external surface of the central open-dome roof as a contour. Similar absolute values were obtained for both open ratios in all the regions.

### 3.4. Internal Peak Pressure Coefficients on the Roof Surface

**Figure 14:**
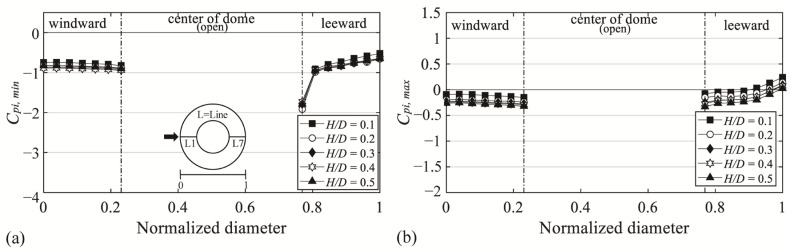
Variation of *C_pi,min_* and *C_pi,max_* depending on *H*/*D* of the central open dome: (**a**) *C_pi,min_* of open ratio = 50% and (**b**) *C_pi,max_* of open ratio = 50%.

Figure 14a shows the negative internal peak pressure coefficient (*C_pi,min_*) on the interior roof surface for an open ratio of 50%. The absolute values are large, owing to the effect of separation at the roof edge of the leeward open space, as in the case of the external surface. The *C_pi,min_* ranges from −1.7 to −1.9, which is smaller than those of the external surface. In other areas, the *C_pi,min_* ranges from −0.8 to −1.0. Figure 14b shows the positive internal peak pressure coefficient (*C_pi,max_*). The coefficient is affected by the positive pressure in the normalized diameter range of approximately 0.9–1.0. This is assumed to occur because, as the open ratio increases, the area in the roof decreases and the amount of incoming air increases, directly affecting the flow on the roof surface of the corresponding region. However, for the interior surface, the negative pressure is dominant; thus, the values are not large.

### 3.5. Comparison of the External Peak Pressure Coefficient of the Edge Open Dome

**Figure 15:**
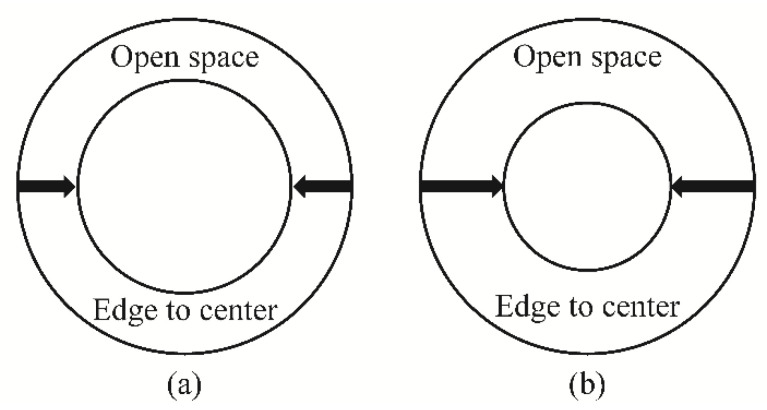
Type of edge open domes (Kim et al., 2019 [19]): (**a**) open ratio = 30% and (**b**) open ratio = 50%.

**Figure 16:**
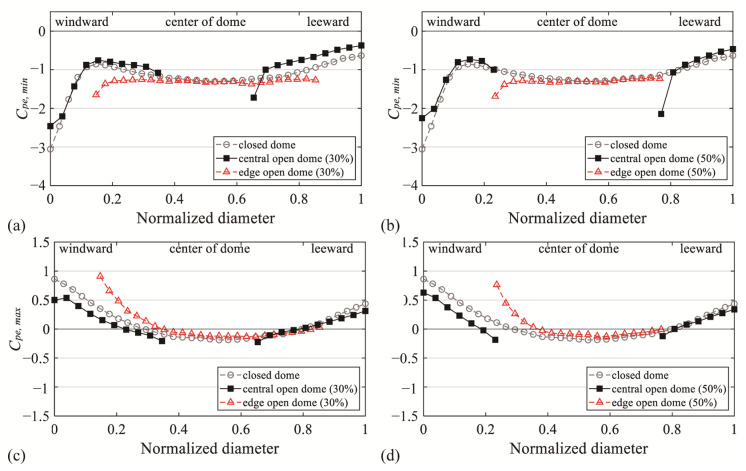
Comparison with edge open domes: (**a**) *C_pe,min_* of *H*/*D* = 0.5 (open ratio = 30%), (**b**) *C_pe,min_* of *H*/*D* = 0.5 (open ratio = 50%), (**c**) *C_pe,max_* of *H*/*D* = 0.1 (open ratio = 30%), and (**d**) *C_pe,max_* of *H*/*D* = 0.1 (open ratio = 50%).

To analyze the difference in the pressure distribution for each open type of dome roof, the model of the edge open dome roof and the peak pressure coefficient for each type are shown in Figure 15 and Figure 16, respectively. For the data on the edge open dome, the results of Kim et al. [20] were used. The open ratio was defined based on the span length *D* for both the open edge and the center open dome.

In the peak pressure coefficients for each open dome type in Figure 16, with reference to *H*/*D* showing the largest absolute values, *C_pe,min_* is presented for *H*/*D* = 0.5, and *C_pe,max_* is presented for *H*/*D* = 0.1. The external peak pressure coefficients for the closed dome roof are also shown in the graph. As described earlier, for *C_pe,min_* in Figure 16a,b, the flow deviated by the open space separated again, increasing the effect of negative pressure compared with the closed dome type. Conversely, for an edge open dome, there is no direct effect from the vortices due to the separation because of the open space, and the absolute values are reduced compared with the closed dome type because of the complicated turbulence of the flow separated from the windward wall. For *C_pe,max_* in Figure 16c,d, the central and edge open domes showed similar absolute values to those of the closed dome type. Based on these results, the edge open dome type is more advantageous against wind loads based on the external surface of the roof.

### 3.6. Comparison with AIJ-RLB (2015)

In terms of closed dome roofs, the Japanese wind load code (AIJ-RLB (2015)) has more detailed values than other wind load codes. Table 3, Table 4 and Table 5 list the peak pressure coefficients for the external cladding for the dome roofs of the ASCE7-16 code in the United States and the AIJ-RLB (2015) code in Japan. For ASCE7-16, the peak pressure coefficients for each degree are presented. No peak pressure coefficient was applicable to dome roofs with *f*/*D* = 0.2 or less. On the contrary, AIJ-RLB (2015) presents wind pressure coefficients in detail in three areas for various *f*/*D* and *H*/*D*. Therefore, the AIJ-RLB (2015) code was used to compare the experimental values.

The Japanese wind load codes are based on the wind tunnel test results from Noguchi and Uematsu [2], and their experimental conditions are listed in Table 6. For comparison, the experimental conditions of the present study are also listed in the table.

The Japanese wind load code classifies the dome roof surface into three regions, *R_a_*, *R_b_*, and *R_c_*, considering the change in wind pressure due to separation and provides the values of the external peak pressure coefficient for each classified region. The *R_a_* and *R_b_* regions are defined as *D*/8, and the *R_c_* region is defined as *D*/2.

**Figure 17:**
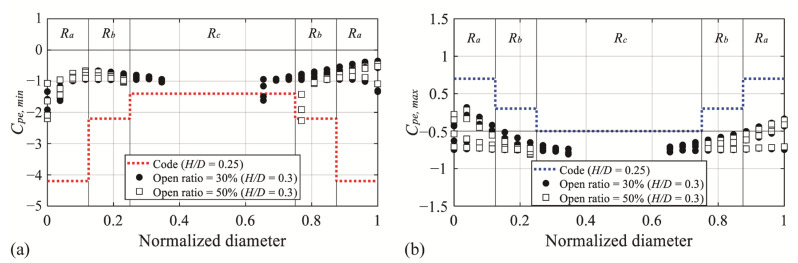
Comparison of the external peak pressure coefficients of AIJ-RLB (2015): (**a**) *C_pe,min_* and (**b**) *C_pe,max_*.

Figure 17 shows the results of the comparison of the external peak pressure coefficients. The dotted line in the graph indicates the external peak pressure coefficient for each region of the code, and the markers represent the experimental values for all wind pressure taps with open ratios of 30% and 50% and an *H*/*D* value of 0.3, similar to the code. For *C_pe,min_* in Figure 17a, the experimental values in the *R_a_* and *R_b_* regions show a significant difference from the code values. As listed in Table 7, this result is because of the differences in the oncoming flow and model dimensions [20]. Conversely, the experimental values exceed the code values in the region (*R_b_* and *R_c_*) where the absolute values increase, owing to the separation of the deviated flow. For *C_pe,max_* in Figure 17b, unlike *C_pe,min_*, the code and experimental values do not show any notable difference, and the experimental values satisfy the code values.

**Figure 18:**
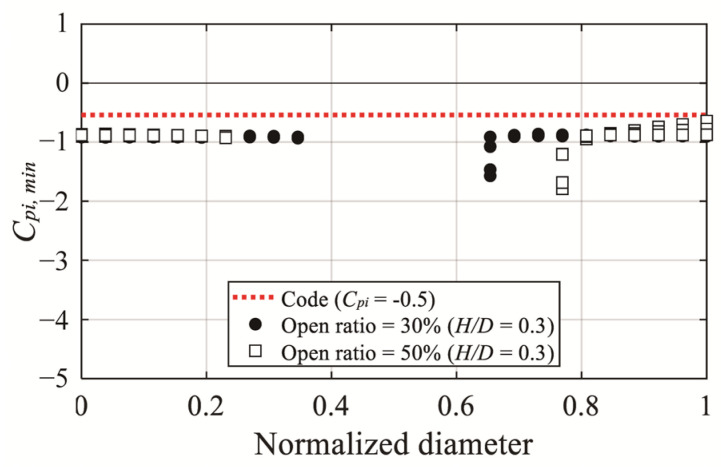
Comparison of the negative internal peak pressure coefficients of AIJ-RLB (2015).

Figure 18 compares the internal peak pressure coefficients of the experiment and the internal pressure coefficient of the code. For the internal pressure coefficient of the code, based on the closed dome roof, a value of zero is suggested for cases with no openings in the wall, and −0.5 is suggested for cases with openings in the wall. Thus, the comparison was made only for *C_pi,min_*. Based on the comparison, the experimental values were larger than those of the code in all regions, and a difference of up to 3.8 times was shown in the region affected by separation. 

The wind loads for the cladding design prescribed by AIJ-RLB (2015) are calculated by the following equation [22]:

(4) $$W_{C}=q_{H}C_{c}A_{C},\;$$

where *W_c_* is the wind load, *q_H_* is the design velocity pressure, *C_c_* is defined as the difference between the external and internal pressure coefficients (*C_pe,peak_*—*C_pi_*), *A_c_* is the subject area of cladding. For the wind loads of the cladding design, two different cases should be considered: (1) a negative pressure on the external roof surface and a positive pressure on the internal roof surface; and (2) a positive pressure on the external roof surface and a negative pressure on the internal roof surface.

The net pressure coefficient—the difference between the external and internal pressures of the central open dome roof—was calculated for comparison. The net pressure coefficients (*C_pn,i_*) are defined as:

(5) $$C_{Pn,i}=C_{pe,i}-C_{pi,i},\;$$

where *C_pe,i_* and *C_pi,i_* are the pressures coefficients calculated by Equation (1) at each pressure tap installed in the same line and location on the external and internal roof surfaces.

(6) $$C_{Pn,mean}=\frac{1}{N}\sum_{i=1}^{N}C_{pn,i}.$$

The mean net pressure coefficients (*C_pn,mean_*) were calculated using Equations (6), and the peak net pressure coefficients (*C_pn,min_* and *C_pn,max_*) were defined as the minimum and maximum values for each *C_pn,i_*.

**Figure 19:**
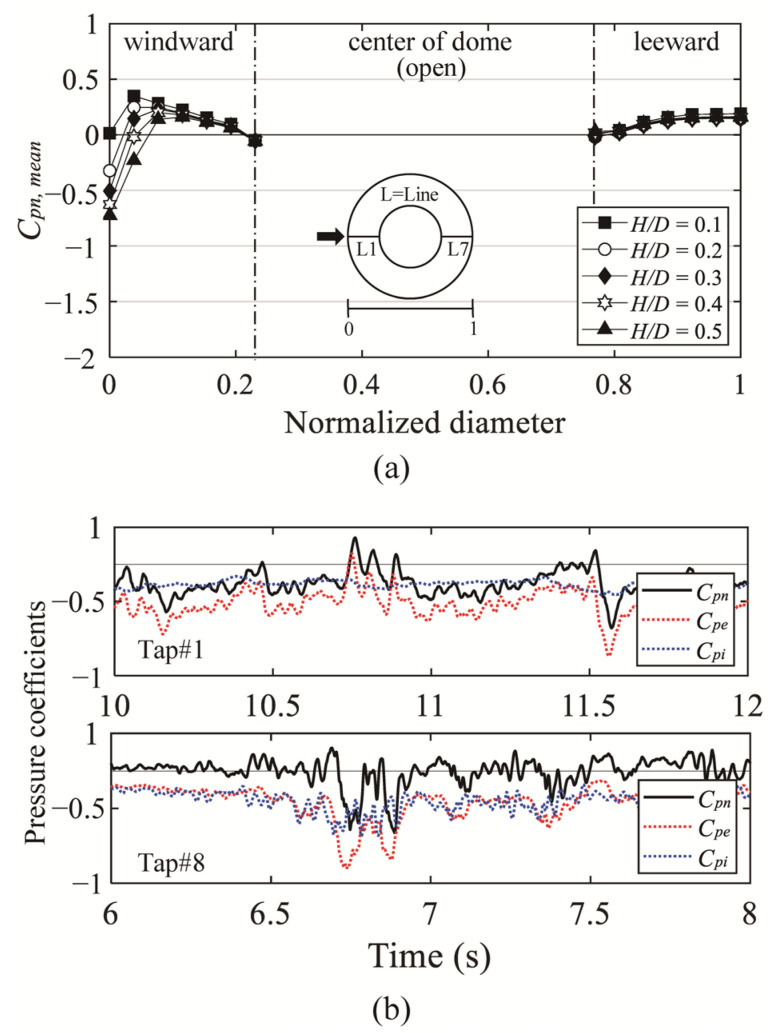

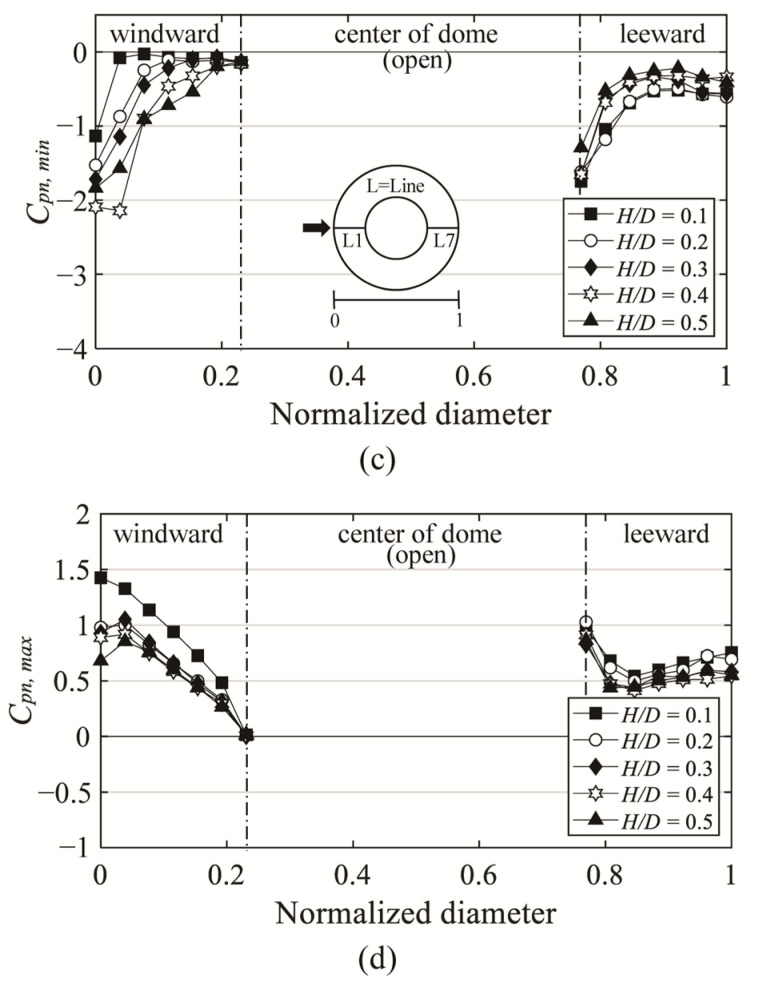
Variation of *C_pn,mean_*, *C_pn,min_*, and *C_pn,max_* depending on *H*/*D* and pressure time histories: (**a**) *C_pn,mean_*; (**b**) time histories of external, internal, and net pressures; (**c**) *C_pn,min_*; and (**d**) *C_pn,max_*.

Figure 19a shows mean net pressure coefficients (*C_pn,mean_*) for an open ratio of 50%. *C_pn,mean_* decrease negative pressure and increase positive pressure compared to *C_pe,mean_*, due to the simultaneous contribution of pressure on the external and internal surfaces of the roof. The internal surface of the roof is only affected by negative pressure. Thus, the negative pressures at the location affected by the separation and boundary layers of the dome surface in the windward region are reduced. Since the wind pressure inside the roof is similar regardless of *H*/*D*, the *C_pn,mean_* changes depending on the *C_pe,mean_*, which is affected by the characteristics of the oncoming flow. However, the *C_pn,mean_* of leeward side are similar in absolute values regardless of *H*/*D* and are close to zero. This is because the external and internal pressures are similar. Figure 19b shows a time series of pressure coefficients. As can be seen from the time series in Tap#1, the *C_pi_* on the windward side are constant, and *C_pn_* are affected by *C_pe_*. However, the leeward side Tap#8 shows very similar *C_pe_* and *C_pi_*. Figure 19c and d shows the *C_pn,min_* and *C_pn,max_* for an open ratio of 50%. The absolute values of the windward side are varied depending on the *H*/*D*, but the leeward side shows very similar absolute values. Compared to the external peak pressure coefficients (*C_pe,min_* and *C_pe,max_*), the absolute values of *C_pn,min_* decreased in all areas, but *C_pn,max_* increased. The greatest increase can be seen especially at the edge of the roof in the open space (see Figure 12).

**Figure 20:**
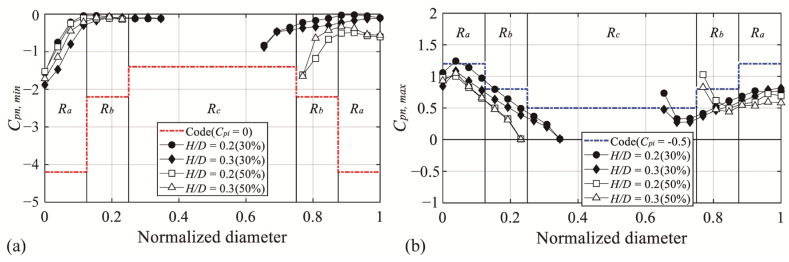
Comparison of the peak net pressure coefficients of AIJ-RLB (2015): (**a**) *C_pn,min_* and (**b**) *C_pn,max_*.

Figure 20 shows a comparison between the experimental value of the peak net pressure coefficient and the code. Figure 20a shows the case with negative pressure on the external surface and positive pressure on the internal surface (Case 1). The experimental values satisfy the code values owing to the overall reduction in the negative pressure by the large effect of negative pressure on the interior surface. The case where there is positive pressure on the external surface and negative pressure on the internal surface (Case 2) is shown in Figure 20b. In contrast to Case 1, the experimental values exceed the code values in all areas. For a clearer comparison, the code and experimental values for each area are summarized in Table 7, Table 8, Table 9 and Table 10. Table 7 and Table 8 list the maximum values of the code and experimental values (*C_pn,min_*) for each area with an open ratio of 30% and 50%. As described above, both open ratios satisfied the codes. Table 9 and Table 10 compare the code and the experimental values (*C_pn,max_*), and both open ratios exceed the codes at the roof edge of the open space. These results indicate that for domes with central openings, wind loads based on the current code may be underestimated or overestimated.

**Table 7:** Comparison of code value according to code area and *C_pn,min_* of open ratio = 30% [22].

*H*/*D*	Code Value (*H*/*D* = 0.25)			Experimental Value		
	*R_a_*	*R_b_*	*R_c_*	*R_a_*	*R_b_*	*R_c_*
0.2	−4.2	−2.2	−1.4	−1.6	−0.2	−0.9
0.3				−1.9	−0.4	−0.9

### 3.7. Proposal of Peak Net Pressure Coefficients for the Central Open Dome 

**Figure 21:**
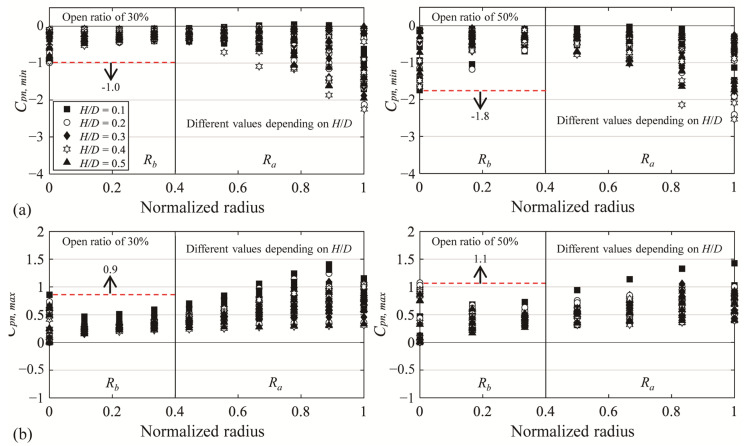
Proposed peak net pressure coefficients for the central open dome: (**a**) *C_pn,min_* and (**b**) *C_pn,max_*.

As seen previously, relatively large changes were found in the peak pressure coefficients on the domes with an opening in the center. When comparing the experimental values and code values, the experimental values exceeded the code values in some regions. Considering those findings, the roof was divided into two zones—*R_a_* and *R_b_*—and peak net pressure coefficients for cladding design were proposed for each zone. The proposed values selected the largest absolute value that appears in each zone. *R_a_* covered the area along the roof edge where large absolute values due to separation of oncoming flow were shown, and *R_b_* covered the area where the absolute values were large due to the separation of the flow deviated from the windward side. To determine a suitable boundary between *R_a_* and *R_b_*, changes in the peak net pressure coefficients were carefully investigated for all *H*/*D*. The boundaries of the two zones were determined by the location where absolute values appear smallest without being affected by separation. Thus, *R_a_* was defined as 60% of the radius of the roof from the roof edge, while *R_b_* was defined as the remaining area. This can be seen in Figure 21.

Figure 21 shows *C_pn,min_* and *C_pn,max_*, which shows the proposed values in the *R_b_* and the experimental values of all pressure taps by *H*/*D*. The x-axis shows the normalized radius defined based on the radius of the dome (except for the open area) and the distance of the pressure taps, “1” indicate the edge of the roof. The dotted lines indicate the proposed values, which are based on the largest absolute values in the applicable region, and the markers indicate the experimental values. With reference to the region affected by separation of the deviated flow, the absolute values are similar irrespective of the *H*/*D* change. Therefore, the *R_b_* region was proposed as a single value. For *C_pn,min_*, a value of −1.0 was proposed for the open ratio of 30%, and for the open ratio of 50%, a value of −1.8 was proposed. In addition, for *C_pn,max_*, values of 0.9 and 1.1 were proposed for the open ratio of 30% and 50%, respectively. The values of the Ra region are different according to *H*/*D*, so it is not expressed in the graph. proposed values are listed in Table 11 and Table 12. (Refer to Figure 1 for region division.)

## 4. Conclusions

In this study, wind pressure characteristics for the central open dome were analyzed via wind tunnel tests, and the applicability of the wind load code was examined based on a comparison with the Japanese wind load code. In addition, based on the analysis and comparison results, the peak net pressure coefficients for the cladding design of the central open dome were proposed. The results further demonstrated the effect of wind pressure on open or retractable dome roofs and provided useful information in terms of actual open and retractable dome roof design and operation. The findings of this study are summarized as follows:

(1) Based on the analysis of the peak pressure coefficients, because of the open space in the center of the dome, the flow that deviated from the windward region was separated from the external and internal surfaces of the dome roof edges of the open space, increasing the impact of negative pressure.
(2) In the case of internal peak pressure coefficients, negative pressure was dominant, and the coefficients were not significantly affected by changes in the wall height–span ratio. This tendency locally increased the negative peak net pressure, in which the load acts in the upward direction, and relatively significantly increased the positive peak net pressure, in which the load acts in the downward direction. In particular, the positive peak net pressure at the edge of the roof in the open space increased the most.
(3) When comparing the experimental values with the wind load code values, the region affected by the separation of the deviated flow showed larger experimental values than the code values. Therefore, the calculation of the wind loads using the current wind load code could result in underestimated values.
(4) Based on the experimental results, peak net pressure coefficients for cladding design were proposed, divided into two zones of *R_a_* and *R_b_*. *R_a_* is the region affected by the separation of the oncoming flow and is affected by the *H*/*D* change. Therefore, various values for each *H*/*D* were proposed for the corresponding region. *R_b_* is the region affected by the separation of the flow deviated from the windward side, and since the values were similar in all *H*/*D*, a single value was proposed. For the negative peak net pressure coefficient, a value of −1.0 was proposed for the open ratio of 30% and, for the open ratio of 50%, a value of −1.8 was proposed. In addition, for the positive net peak pressure coefficient, values of 0.9 and 1.1 were proposed for the open ratio of 30% and 50%, respectively.

## Figures and Tables

**Table 1:** Model dimensions.

*f* (in Full Scale, m)	*H* (in Full Scale, m)	*D* (in Full Scale, m)	*f*/*D*	*H*/*D*
0.04 (6)	0.04 (6)	0.4 (60)	0.1	0.1
	0.08 (12)			0.2
	0.12 (18)			0.3
	0.16 (24)			0.4
	0.2 (30)			0.5

**Table 2:** Experimental conditions.

Conditions	Value
Length scale	1/150
Velocity scale	1/3
Time scale	1/50
Wind direction	0°
10 min sample number	10
Sampling frequency	1000 Hz
Moving average time	1 s

**Table 3:** External peak pressure coefficients for cladding design prescribed ASCE7-16 [21].

External Peak Pressure Coefficients for Domes with a Circular Base			
**θ, degrees**	**Negative Pressures**	**Positive Pressures**	**Positive Pressures**
	0–90	0–60	61–90
GC_p_	−0.9	+0.9	+0.5

**Table 4:** Negative peak pressure coefficients for cladding design prescribed in AIJ-RLB (2015) [22].

*f*/*D*	Zone *R_a_*			Zone *R_b_*			Zone *R_c_*		
	*H*/*D*			*H*/*D*			*H*/*D*		
	0	0.25	1	0	0.25	1	0	0.25	1
0	−4.4	−5.1	−3.3	−1.5	−3.7	−3.0	−0.4	−2.3	−2.3
0.05	−3.0	−4.8	−3.3	−1.5	−2.7	−2.7	−1.3	−1.3	−1.3
0.1	−2.0	−4.2	−3.0	−1.5	−2.2	−2.2	−1.4	−1.4	−1.4
0.2	−2.0	−2.0	−2.0	−1.9	−1.9	−1.9	−2.1	−2.1	−2.1
0.5	−2.6	−2.6	−2.6	−2.8	−2.8	−2.8	−3.0	−3.0	−3.0

**Table 5:** Positive peak pressure coefficients for cladding design prescribed in AIJ-RLB (2015) [22].

*f*/*D*	Zone *R_a_*			Zone *R_b_*			Zone *R_c_*		
	*H*/*D*			*H*/*D*			*H*/*D*		
	0	0.25	1	0	0.25	1	0	0.25	1
0	0.6	0.4	0.4	1.1	0.5	0.5	0.6	0.6	0.6
0.05	1.3	0.5	0.5	1.0	0.4	0.4	0.5	0.1	0.1
0.1	1.7	0.7	0.7	0.9	0.3	0.3	0.4	0	0
0.2	0.9(1 + 7*I_H_*)	0.6(1 + 7*I_H_*)	0.4(1 + 7*I_H_*)	1.2	0.6	0.6	0.2	0	0
0.5	1 + 7*I_H_*	1 + 7*I_H_*	1 + 7*I_H_*	1.9	1.3	0.7	0.3	0	0

**Table 6:** Comparison of the experimental conditions [2].

	Noguchi and Uematsu [2003]	This Study
α	0.15 and 0.27	0.21
D (m)	0.267	0.4
Moving average	1 s	1 s

**Table 8:** Comparison of code value according to code area and *C_pn,min_* of open ratio = 50% [22].

*H*/*D*	Code Value (*H*/*D* = 0.25)			Experimental Value		
	*R_a_*	*R_b_*	*R_c_*	*R_a_*	*R_b_*	*R_c_*
0.2	−4.2	−2.2	−1.4	−1.5	−1.7	-
0.3				−1.7	−1.7	-

**Table 9:** Comparison of code value according to code area and *C_pn,max_* of open ratio = 30% [22].

*H*/*D*	Code Value (*H*/*D* = 0.25)			Experimental Value		
	*R_a_*	*R_b_*	*R_c_*	*R_a_*	*R_b_*	*R_c_*
0.2	1.2	0.8	0.5	1.2	0.8	0.8
0.3				1.0	0.6	0.5

**Table 10:** Comparison of code value according to code area and *C_pn,max_* of open ratio = 50% [22].

*H*/*D*	Code Value (*H*/*D* = 0.25)			Experimental Value		
	*R_a_*	*R_b_*	*R_c_*	*R_a_*	*R_b_*	*R_c_*
0.2	1.2	0.8	0.5	1.0	1.0	-
0.3				1.0	0.9	-

**Table 11:** Proposed negative peak net pressure coefficients.

*H*/*D*	*f*/*D* = 0.1			
	*R_a_* (0.6 *d*)		*R_b_* (0.4 *d*)	
	*r*/*D* = 0.3	*r*/*D* = 0.5	*r*/*D* = 0.3	*r*/*D* = 0.5
0.1	−2.0		−1.0	−1.8
0.2	−2.3			
0.3	−2.3			
0.4	−2.4			
0.5	−2.1			

**Table 12:** Proposed positive peak net pressure coefficients.

*H*/*D*	*f*/*D* = 0.1			
	*R_a_* (0.6 *r*)		*R_b_* (0.4 *r*)	
	*r*/*D* = 0.3	*r*/*D* = 0.5	*r*/*D* = 0.3	*r*/*D* = 0.5
0.1	1.4		0.9	1.1
0.2	1.2			
0.3	1.0			
0.4	1.0			
0.5	1.0			

## Data Availability

The data presented in this study are available on request from the corresponding author.
